# Profiling Tissue and Biofluid miR-155-5p, miR-155^*^, and miR-146a-5p Expression in Graft vs. Host Disease

**DOI:** 10.3389/fimmu.2021.639171

**Published:** 2021-03-15

**Authors:** Rachel E. Crossland, Jean Norden, Sakhila Ghimire, Mateja Kralj Juric, Kim F. Pearce, Clare Lendrem, Matthew Collin, Eva Mischak-Weissinger, Ernst Holler, Hildegard T. Greinix, Anne M. Dickinson

**Affiliations:** ^1^Faculty of Medical Sciences, Translational and Clinical Research Institute, Newcastle University, Newcastle upon Tyne, United Kingdom; ^2^Department of Haematology and Oncology, University of Regensburg, Regensburg, Germany; ^3^Department of Internal Medicine I, Medical University of Vienna, Vienna, Austria; ^4^Department of Haematology, Hemostasis, Oncology and Stem Cell Transplantation, Hannover Medical School, Hanover, Germany; ^5^Division of Hematology, Medical University of Graz, Graz, Austria

**Keywords:** microRNA, GvHD, biomarker, transplantation, extracellular vesicle

## Abstract

**Introduction:** Acute graft vs. host disease (aGvHD) is a frequent complication following allogeneic haematopoeitic transplantation (HSCT). Despite recent advances, there are no universally accepted biomarkers to determine development of aGvHD. MicroRNAs miR-146a and miR-155 have been previously associated with aGvHD and show promise as clinically translatable biomarkers. In this study, we performed comprehensive expression profiling of miR-146a, miR-155, and miR-155^*^ expression in aGvHD target tissue and biofluids and relate expression to post-HSCT outcomes.

**Materials and Methods:** MicroRNA expression was assessed by qRT-PCR in gastrointestinal (*n* = 31) and skin (*n* = 31) biopsies as well as serum (exploratory cohort *n* = 34, verification cohort *n* = 81, diagnostic cohort *n* = 65) and urine (exploratory cohort *n* = 30, verification cohort *n* = 56, diagnostic cohort *n* = 20) biofluids, including extracellular vesicle (EV) cohorts (serum EV *n* = 15, urine EV *n* = 30). Expression was related to aGvHD incidence, severity and overall survival.

**Results:** In GI samples, expression of miR-155 (*p* = 0.03) and miR-146a (*p* = 0.03) was higher at aGvHD onset compared to patients with no GvHD. In skin biopsies, expression of miR-155 (*p* = 0.004) was upregulated in aGvHD patients compared to normal control skin. Expression of miR-146a was higher in aGvHD compared to no aGvHD biopsies (*p* = 0.002). In serum, miR-155 (*p* = 0.03) and miR-146a (*p* = 0.02) expression was higher at day 14 (D14), while in urine expression was elevated at D7 post-HSCT in patients who developed aGvHD compared to those disease-free. This was verified in an independent serum (miR-155 *p* = 0.005, miR-146a *p* = 0.003) and urine (miR-155 *p* = 0.02, miR-146a *p* = 0.04) cohort, where both microRNAs were also associated with aGvHD by ROC analysis. In serum and urine samples taken at the time of aGvHD symptoms, expression of miR-155 and miR-146a was also elevated (serum miR-155 *p* = 0.03, miR-146a *p* < 0.001; urine miR-155 *p* = 0.02, miR-146a *p* = 0.02). In contrast, miR-146a and miR-155 were downregulated at D14 in serum EVs and at D7 in urine EVs in patients who developed aGvHD compared to those that remained disease-free, in both an exploratory (serum miR-155 *p* = 0.02, miR-146a *p* = 0.06; urine miR-155 *p* = 0.02, miR-146a *p* = 0.07) and an independent cohort (serum miR-155 *p* = 0.01, miR-146a *p* = 0.02).

**Conclusions:** These results further support a role for miR-155 and miR-146a as non-invasive, clinically relevant biomarkers for aGvHD. However, the link between their involvement in generalized inflammation and in specific pathophysiology requires further investigation at a systemic level.

## Introduction

Allogeneic hematopoietic stem cell transplantation (allo-HSCT) is the treatment of choice for many types of hematological malignancy and autoimmune disorders. However, despite recent advances the mortality rate remains high at around 40–60%, chiefly due to post-transplant complications including acute and chronic graft vs. host disease (GvHD), diease progression/relapse and opportunistic infections (1). GvHD occurs within the skin, gut and liver in approximately 40–70% of HSCT patients and acute GVHD (aGvHD) develops in the first 3 months post-transplant, with a mortality of around 30% (2). High resolution human leukocyte antigen (HLA) matching has improved HSCT outcome over recent years, however, aGvHD still remains a serious complication. There is a need for specific novel and robust universally accepted biomarkers that can be used in the clinic to predict aGvHD incidence and severity. In addition, these markers may also be targeted as novel therapeutics to prevent GvHD incidence and improve patient overall survival.

MicroRNAs have been shown to play an important role in the modulation of gene expression (3). MicroRNAs are a class of non-coding RNAs, approximately 19-22 nucleotides in length. To date, over a thousand microRNAs have been identified and they comprise between 1 and 5% of all genes in the human genome. They function by targeting the 3′ un-translated region (UTR) of mRNAs, by binding to complementary seed sequences. MicroRNAs play an important role in post transcriptional gene regulation by either translation repression, or less frequently, by mRNA degradation (4). More recently, microRNAs have been shown to be present in biofluids, where they are protected from RNase mediated degradation by encapsulation into extracellular vesicles (EVs) or through binding to protective proteins [reviewed in (5)]. This novel mode of intercellular communication allows microRNAs to be transported to distal target cells, where they have biological function (6, 7). It has been shown EVs from both non-immune and immune cells have important roles in immune regulation (8). The role of EVs in the pathophysiology of immunological disorders, including GvHD, is an exciting field of research, as EVs offer importance as prospective therapeutic targets, informative biological agents and predictive disease biomarkers (9).

MiR-155 has been directly implicated in the control of immunity, by modulating both the adaptive and innate immune response (10). Encoded within a region known as the B-cell integration cluster (BIC), miR-155 has been implicated in the pathogenesis of a number of autoimmune diseases, including rheumatoid arthritis (RA) and systemic lupus erythematosus (SLE). More recently, miR-155 has been directly implicated in aGvHD, whereby Ranganathan et al. found that miR-155 was up regulated in the T-cells of mice with aGvHD (11). Moreover, the use of miR-155 inhibitors decreased disease severity and prolonging survival (11). Expression of miR-155 was reported to be regulated in small and large bowel biopsies taken from a cohort of patients with intestinal aGvHD in comparison to normal bowel tissue (11), and subsequent studies assessing miR-155 as a biofluid biomarker for aGvHD have also shown promise (12–14).

During microRNA biogenesis, the abundant strand is frequently referred to as the mature microRNA (miR-5p) and is selectively loaded onto the RNA-induced silencing complex (RISC). The star form (miR-^*^/miR-3p) strand was originally thought to be non-functional and degraded. However, it has been reported that the 2 forms of miR-155 (miR-155/miR-155^*^) act antagonistically on the expression of interferon alpha (IFNα) in plasmacytoid dendritic cells (15). In this context, miR-155 targets TGF-Beta Activated Kinase 1/MAP3K7 Binding Protein 2 (TAB2) resulting in decreased IFNα, while miR-155^*^ targets interleukin 1 receptor associated kinase 1 (IRAK1) leading to increased IFNα, highlighting the importance of the star form depending on the biological setting.

MicroRNA-146 also plays a key role in the regulation of innate and adaptive immunity. It's expression is induced in response to microbial components such as lipopolysaccharide (LPS), which can trigger GvHD pathology (16, 17). Stickel et al. reported that miR-146a expression is upregulated in the T-cells of mice that develop aGvHD compared to untreated mice (17). Transplantation of miR-146a^−/−^ T-cells resulted in reduced survival, upregulation of TRAF6 and TNF expression and increased aGvHD severity (17). In addition, treatment of aGvHD model mice with a miR-146a mimic results in an IFN-γ burst, that leads to reduced LPS-induced TNFa release and GvHD-associated weight loss, as well as prolonged survival (18).

Our group has previously shown that miR-146a-5p and miR-155-5p expression levels in the peripheral blood of allogeneic-HSCT (allo-HSCT) patients post-transplant can be predictive of aGVHD incidence (14, 19). Considering the role of miR-155 and miR-146a in aGvHD pathology, this study sought to comprehensively profile the expression of miR-155, miR-155^*^, and miR-146a in a range of clinical samples taken from patients undergoing allo-HSCT, including EV fractions, and relate expression to aGvHD incidence and severity, as well as post-HSCT outcomes. Expression profiling was performed in HSCT patient body fluids including serum and urine, in the EV fraction of serum and urine and in tissue samples including the skin and gastrointestinal tract. Results will improve our understanding of the role of miR-155, miR-155^*^, and miR-146a in the pathophysiology of GvHD, as well as further explore their potential for biomarker utility.

## Materials and Methods

### Patient Cohorts and Sample Collection

Gastrointestinal (GI) tissue biopsies from (*n* = 31) HSCT patients transplanted between 2009 and 2013 at the University Medical Centre Regensburg (Regensburg, Germany) were obtained during a screening program to detect early GvHD or at the onset or persistence of clinical symptoms of GI GvHD (Table 1). Following endoscopy, GI biopsies were collected in RNA*later*® (Ambion) for subsequent RNA isolation and microRNA analysis. Biopsies were also collected in formalin followed by paraffin embedding for the purpose of scoring GvHD stage according to the Lerner system, by histopathologists blinded to the overall clinical grading (20). Skin biopsies from were taken from normal controls (*n* = 6), pre-transplant (*n* =6), and post-HSCT (*n* = 31) patients undergoing HSCT between 2011 and 2012 at the Newcastle Freeman Hospital (Newcastle upon Tyne, UK) and stored at −80°C in RNA*later*® (Ambion) prior to processing. The clinical aGvHD biopsies were taken between 16 and 50 days post-transplant at the time of clinical aGvHD diagnosis, according to standard criteria (21) (Table 1).

**Table 1:** Clinical characteristics for tissue biopsy patient samples.

	**Skin cohort**				**Gastrointestinal cohort**			
**Characteristic**	**All**	**aGvHD (*N*, %)**	**No GvHD (*N*, %)**	***p*-value**	**All**	**aGvHD (*N*, %)**	**No GvHD (*N*, %)**	***p*-value**
Patients	31	27	4		31	16	15	
**Age**								
Average	50	50	56	0.44	49	53	46	0.20
Range	28–66	28–66	43–63		17–66	17–64	17–66	
**Gender**								
Male	24	20 (74)	4 (100)	0.55	21	11 (69)	10 (67)	1.0
Female	7	7 (26)	0 (0)		10	5 (31)	5 (33)	
**Diagnosis**								
AML	11	10 (37)	1 (25)	0.36	15	8 (50)	7 (46)	0.91
N-HL	8	7 (26)	1 (25)		3	2 (12)	1 (7)	
CLL	1	0 (0)	1 (25)		3	2 (12)	1 (7)	
ALL	6	5 (19)	1 (25)		2	1 (7)	1 (7)	
MDS	1	1 (4)	0 (0)		5	2 (12)	2 (12)	
PCN	1	1 (4)	0 (0)		1	0 (0)	0 (0)	
CM	1	1 (4)	0 (0)		0	0 (0)	0 (0)	
HD	2	2 (6)	0 (0)		1	0 (0)	1 (7)	
AA	0	0 (0)	0 (0)		1	0 (0)	1 (7)	
MM	0	0 (0)	0 (0)		0	1 (7)	1 (7)	
**Conditioning**								
Myeloablative	6	5 (19)	1 (25)	1.0	4	3 (19)	1 (7)	0.60
RIC	25	22 (81)	3 (75)		27	13 (81)	14 (93)	
**Prophylaxis**								
CyA + MTX	31	27 (100)	4 (100)	1.0	26	11 (69)	15 (100)	0.42
CyA + MMF	0	0 (0)	0 (0)		2	2 (13)	0 (0)	
Tacrolimus +/– MTX	0	0 (0)	0 (0)		3	3 (19)	0 (0)	
**Relation**								
MUD	25	22 (81)	3 (75)	1.0	23	13 (81)	10 (67)	0.43
SIB	6	5 (19)	1 (25)		8	3 (19)	5 (33)	
**CMV status**								
Positive	12	8 (30)	4 (100)	0.02	13	5 (31)	8 (53)	0.29
Negative	19	19 (70)	0 (0)		18	11 (69)	7 (47)	
**Survival**								
Alive	18	14 (52)	4 (100)	0.12	16	12 (75)	4 (27)	0.01
Deceased	13	13 (48)	0 (0)		15	4 (25)	11 (73)	

*Clinical information for the collective cohorts as well as within aGvHD and no GvHD sub-groups are shown. Differences in clinical characteristics between aGvHD vs. no GvHD groups (according to histopathological stage) were calculated using Fisher's exact test or the Student's t-test, as appropriate. AML, Acute myeloid leukemia; N-HL, Non-Hodgkin's lymphoma; CLL, Chronic lymphocytic leukemia; ALL, Acute lymphoblastic leukemia; MDS, Myelodysplastic syndrome; PCN, Plasma cell neoplasm; CM, Chronic Myeloproliferation; HD, Hodgkin's disease; RIC, reduced intensity conditioning; CyA, Cyclosporine A; MTX, Methotrexate; MMF, Mycophenolate Mofetil; MUD, matched unrelated donor; SIB, sibling donor; CMV, Cytomegalovirus*.

Sera were collected at sequential time points (D-7, D0, D7, D14, D28) using vacutainers with no coagulant. They were left to clot, centrifuged at 500 g for 5 min and the supernatant removed and stored at −80°C. The sequential sample exploratory cohort comprised of *n* = 34 patients transplanted between 2009 and 2013, and the verification cohort included *n* = 81 patients transplanted between 2009 and 2013 at the Newcastle Freeman Hospital (Newcastle upon Tyne, UK) (Table 2). Results were further verified in an independent diagnostic cohort collected from *n* = 65 patients at the time of aGvHD onset, from the Medical University Vienna (Vienna, Austria) undergoing HSCT between 2007 and 2014 (Table 2).

**Table 2:** Clinical characteristics for serum biofluid patient samples.

	**Serum exploratory cohort**				**Serum verification cohort**				**Serum diagnostic cohort**			
**Characteristic**	**All**	**aGvHD** ** (*N*, %)**	**No GvHD** ** (*N*, %)**	***p*-value**	**All**	**aGvHD** ** (*N*, %)**	**No GvHD** ** (*N*, %)**	***p*-value**	**All**	**aGvHD** ** (*N*, %)**	**No GvHD** ** (*N*, %)**	***p*-value**
**Patients**	34	20	14		81	44	37		65	41	24	
**Age**												
Average	48	43	56	0.07	49	46	53	0.09	47	45	49	0.33
Range	20–67	20–67	41–63		20–49	20–69	20–69		18–73	18–73	19–71	
**Gender**												
Male	25	14 (70)	11 (79)	0.53	48	27 (61)	21 (57)	0.82	36	26 (63)	10 (42)	0.12
Female	9	6 (30)	3 (21)		33	17 (39)	16 (43)		29	15 (37)	14 (58)	
**Diagnosis**												
MDS	12	8 (40)	4 (20)	0.52	9^*^	5 (18)	4 (15)	0.85	4^*^	4 (10)	0 (0)	0.67
N-HL	8	5 (25)	3 (15)		15^*^	8 (30)	7 (25)		4^*^	2 (5)	2 (12)	
AML	5	2 (10)	3 (15)		12^*^	5 (18)	7 (25)		34^*^	24 (59)	10 (58)	
ALL	4	2 (10)	2 (10)		6^*^	4 (14)	2 (7)		10^*^	7 (17)	3 (18)	
HD	2	2 (10)	0 (0)		2^*^	1 (4)	1 (4)		0^*^	0 (0)	0 (0)	
CLL	1	0 (0)	1 (5)		1^*^	0 (0)	1 (4)		0^*^	0 (0)	0 (0)	
CML	1	0 (0)	1 (5)		2^*^	1 (4)	1 (4)		5^*^	3 (7)	2 (12)	
MM	1	1 (5)	0 (0)		2^*^	1 (4)	1 (4)		0^*^	0 (0)	0 (0)	
Amyloidosis	0	0 (0)	0 (0)		1^*^	0 (0)	1 (4)		0^*^	0 (0)	0 (0)	
CGD	0	0 (0)	0 (0)		1^*^	1 (4)	0 (0)		0^*^	0 (0)	0 (0)	
MPS	0	0 (0)	0 (0)		1^*^	0 (0)	1 (4)		0^*^	0 (0)	0 (0)	
Secondary acute	0	0 (0)	0 (0)		1^*^	1 (4)	0 (0)		0^*^	0 (0)	0 (0)	
Leukemia	0	0 (0)	0 (0)		1^*^	0 (0)	1 (4)		0^*^	0 (0)	0 (0)	
Anaplastic anemia	0	0 (0)	0 (0)		0^*^	0 (0)	0 (0)		1^*^	1 (2)	0 (0)	
T-cell lymphoma												
**Conditioning**												
Myeloablative	6	4 (20)	2 (14)	0.59	20	14 (32)	6 (16)	0.13	25^*^	19 (53)	6 (29)	0.10
RIC	28	16 (80)	12 (86)		61	30 (68)	31 (84)		32^*^	17 (47)	15 (71)	
**Relation**												
MUD	27	9 (50)	11 (79)	0.56	47	26 (59)	21 (57)	1.0	43^*^	31 (78)	12 (60)	0.74
SIB	7	9 (50)	3 (21)		34	18 (41)	16 (43)		14^*^	9 (22)	5 (40)	
**CMV status**												
Positive	15	5 (26)	10 (67)	0.04	37^*^	13 (33)	24 (67)	0.006	27^*^	18 (56)	9 (75)	0.32
Negative	19	14 (74)	5 (33)		39^*^	27 (67)	12 (33)		17^*^	14 (44)	3 (25)	
**Survival**												
Alive	18	8 (40)	10 (71)	0.07	51	25 (57)	26 (70)	0.25	57	35 (85)	22 (92)	0.70
Deceased	16	12 (60)	4 (29)		30	19 (43)	11 (30)		8	6 (15)	2 (8)	

*Clinical information for the collective serum cohorts as well as within aGvHD and no GvHD sub-groups are shown. Differences in clinical characteristics between aGvHD vs. no GvHD groups (according to overall clinical grade) were calculated using Fisher's exact test or the Student's t-test, as appropriate. MDS, Myelodysplastic syndrome; N-HL, Non-Hodgkin's lymphoma; AML, Acute myeloid leukemia; ALL, Acute lymphoblastic leukemia; HD, Hodgkin's disease; CLL, Chronic lymphocytic leukemia; CML, Chronic Myelogenous Leukemia; MM, Multiple myeloma; CGD, Chronic Granulomatous Disease; MPS, Myeloproliferative syndrome; RIC, reduced intensity conditioning; MUD, matched unrelated donor; SIB, sibling donor; CMV, Cytomegalovirus; N/A, Not available*.

* *, Some clinical data unavailable*.

Urine samples were sequentially collected 7 days pre-transplant (D-7), on the day of transplant (D0) and then at sequential time-points post-HSCT (D7, D14). The sequential sample exploratory cohort consisted of *n* = 30 patients transplanted between 2011 and 2013, and the verification cohort comprised *n* = 56 patients transplanted between 2011 and 2013 (Table 3). All samples were obtained from patients at the Newcastle Freeman Hospital (Newcastle upon Tyne, UK), undergoing HSCT between 2011 and 2013 (Table 3). Results were further validated in an independent diagnostic cohort collected from *n* = 20 patients at the time of aGvHD onset from an independent cohort collected from patients transplanted at the Medical University Vienna, or the University Medical Center Regensburg between 2008 and 2011 (Table 3).

**Table 3:** Clinical characteristics for urine biofluid patient samples.

	**Urine exploratory cohort**				**Urine verification cohort**				**Urine diagnostic cohort**			
**Characteristic**	**All**	**aGvHD** ** (*N*, %)**	**No GvHD** ** (*N*, %)**	***p*-value**	**All**	**aGvHD** ** (*N*, %)**	**No GvHD** ** (*N*, %)**	***p*-value**	**All**	**aGvHD** ** (*N*, %)**	**No GvHD** ** (*N*, %)**	***p*-value**
Patients	30	21	9		56	38	18		20	14	6	
**Age**												
Average	52	51	53	0.79	50	49	53	0.62	47	49	45	0.57
Range	20–68	28–67	42–67		(20–67)	(20–67)	(26–67)		(19–64)	(30–58)	(19–64)	
**Gender**												
Male	19	15 (71)	4 (44)	0.23	39	27 (71)	12 (67)	0.76	11	8 (57)	3 (50)	1.0
Female	11	6 (29)	5 (56)		17	11 (29)	6 (33)		9	6 (43)	3 (50)	
**Diagnosis**												
MDS	7	5 (24)	2 (22)	0.45	11	3 (16)	8 (21)	0.25	3	2 (14)	1 (17)	0.87
N-HL	6	3 (14)	3 (34)		7	4 (22)	3 (8)		1	1 (7)	0 (0)	
AML	8	7 (33)	1 (11)		18	4 (22)	14 (37)		8	5 (37)	3 (49)	
ALL	1	1 (5)	0 (0)		7	2 (11)	5 (13)		0	0 (0)	0 (0)	
HD	2	2 (10)	0 (0)		3	0 (0)	3 (8)		1	1 (7)	0 (0)	
CLL	0	0 (0)	0 (0)		0	0 (0)	0 (0)		2	1 (7)	1 (17)	
CML	0	0 (0)	0 (0)		1	1 (6)	0 (0)		1	1 (7)	0 (0)	
MM	0	0 (0)	0 (0)		0	0 (0)	0 (0)		0	0 (0)	0 (0)	
Anaplastic anemia	1	0 (0)	1 (11)		1	1 (6)	0 (0)		0	0 (0)	0 (0)	
PCN	2	1 (5)	1 (11)		5	2 (11)	3 (8)		3	2 (14)	1 (17)	
CMP	3	2 (10)	1 (11)		3	1 (6)	2 (5)		0	0 (0)	0 (0)	
Myeloproliferative neoplasm	0	0 (0)	0 (0)		0	0 (0)	0 (0)		1	0 (0)	0 (0)	
**Conditioning**												
Myeloablative	23	16 (76)	7 (78)	1.0	14	12 (32)	2 (11)	0.18	6	3 (21)	3 (50)	0.30
RIC	7	5 (24)	2 (22)		42	26 (68)	16 (89)		14	11 (79)	3 (50)	
**Relation**												
MUD	20	14 (67)	6 (67)	1.0	37	25 (66)	12 (67)	1.0	14	11 (79)	3 (50)	0.30
SIB	10	7 (33)	3 (33)		19	13 (34)	6 (33)		6	3 (21)	3 (50)	
**CMV status**												
Positive	17	12 (57)	5 (56)	1.0	26	17 (45)	9 (50)	0.78	7	5 (36)	2 (33)	1.0
Negative	13	9 (43)	4 (44)		30	21 (55)	9 (50)		13	9 (64)	4 (67)	
**Survival**												
Alive	19	12 (57)	7 (76)	0.42	33	21 (55)	12 (67)	0.56	N/A	N/A	N/A	N/A
Deceased	11	9 (43)	2 (24)		23	17 (45)	6 (33)					

*Clinical information for the collective urine cohorts as well as within aGvHD and no GvHD sub-groups are shown. Differences in clinical characteristics between aGvHD vs. no GvHD groups (according to overall clinical grade) were calculated using Fisher's exact test or the Student's t-test, as appropriate. MDS, Myelodysplastic syndrome; N-HL, Non-Hodgkin's lymphoma; AML, Acute myeloid leukemia; ALL, Acute lymphoblastic leukemia; HD, Hodgkin's disease; CLL, Chronic lymphocytic leukemia; CML, Chronic myelogenous leukemia; MM, Multiple myeloma; PCN, Plasma cell neoplasm; CMP, Chronic myeloproliferative disease; RIC, reduced intensity conditioning; MUD, matched unrelated donor; SIB, sibling donor; CMV, Cytomegalovirus; N/A, Not available*.

All patients and healthy volunteers consented for sample collection and molecular testing and the project was approved by the Newcastle and North Tyneside Research Ethics Committee (REC Red: 14/NE/1136 and 07/H0906/131), University of Regensburg Ethics Commission (approval no 02/220 and 09/059) and Ethics Committee of the Medical University of Vienna, Austria. All investigations were conducted in accordance with the Helsinki Declaration. The overall clinical aGvHD grade was diagnosed by clinicians in accordance with the NIH consensus and modified Glucksberg criteria (21, 22). All clinical data was collected from the ProMISE database as used by the European Society for Blood and Marrow Transplantation.

### Extracellular Vesicle and RNA Isolation

Total RNA was extracted from GI tissue and skin biopsies using the *mir*VANA miRNA Isolation Kit (Ambion) as per the manufacturer's protocol and RNA purity was measured using NanoDrop 1000 (Thermo Fisher Scientific). For sera samples, total RNA was isolated from 250 μl aliquots using the Norgen Total RNA Isolation Kit (Norgen Biotek), following the manufacturer's instructions. All aliquots were initially centrifuged at 4,500 × g for 15 min to remove platelets. Serum EVs were isolated from 250 μl serum using Life Technologies (LT) Total Exosome Isolation Reagent, according to the manufacturer's protocol, and validated by transmission electron microscopy and nanoparticle tracking analysis, as previously described (23, 24). For RNA isolation from the EV fraction, the EV pellet was initially re-suspended in lysis solution with the addition of 100:1 β-mercaptoethanol. RNA was then isolated using the Norgen Total RNA isolation kit (Norgen Biotek), following the manufacturer's instructions. MicroRNA was isolated from urine using a urine microRNA purification kit (Norgen Biotek) following the manufacturer's protocol. Urine EVs were isolated from 5 ml of urine using a urine exosome isolation kit (Norgen Biotek), as per the manufacturer's instructions.

### cDNA Synthesis and Quantitative RT-PCR

cDNA was generated by reverse transcription of 10 ng of tissue RNA or a standard 5 μl input volume of biofluid RNA using the TaqMan® MicroRNA Reverse Transcription Kit (Life Technologies) and microRNA specific primers provided with the specific Taqman® MicroRNA assays. Real time PCR was carried out using individual Taqman® MicroRNA assays (Life Technologies) for miR-155, miR-155^*^, and miR-146a. Reference small RNAs for data normalization were identified using NormFinder, or according to previous studies (23, 25) and details are given in Supplementary Table 1. Reactions were performed in triplicate using Sensifast^TM^ Probe Hi-ROX × 2 gene expression master mix (Bioline), 5 μl of cDNA and the manufacturer's recommended concentration of Taqman Assay. All reactions were performed μl on a 7900 qRT-PCR system (Life Technologies).

### Statistical Analysis

MicroRNA expression was assessed using SDS2.4 software (Life Technologies) and fold change was determined as for comparative Ct method (26). Statistical analyses were carried out using SPSS v19.0, Sigmaplot v13 or GraphPad Prism v6.0. Differences between groups were assed using the independent *t*-test, one-way ANOVA, or one-way ANOVA repeated measure analysis. Following a significant ANOVA result, differences between pairs of groups were detrmined via the Tukey *post-hoc* test (Prism v6.0). Receiver operating characteristic (ROC) analysis was performed using marker expression on a continuous scale as the test variable and disease status as the state variable (SigmaPlot v13). For the ROC analyses, pre-test prior-probability was set to 0.5 and Cost Ratio to 1.0 (27). The optimal cutoff value to dichotomise microRNA expression was computed from sensitivity and specificity using the slope m by finding the cutoff that maximizes the function: *sensitivity-m(1-specificity)* (SigmaPlot v13) (28). The accuracy of the test was defined by the area under the curve (AUC), whereby AUC = 0.5 means no diagnostic ability and AUC = 1 means perfect diagnostic ability.

## Results

### MiR-155, miR-155^*^, and miR-146a Expression in Gastrointestinal Tissue

Expression of miR-155, miR-155^*^, and miR-146a was assessed in *n* = 31 clinical GI tissue biopsies taken at aGvHD onset or persistence (mean 28 days post-HSCT) [histology stage 1 = 7 (23%), 2 = 8 (26%), 3 = 0 (0%), and 4 = 1 (3%); no aGvHD *n* = 15 (48%)] (Table 1). Patients were of mixed underlying diagnosis, predominantly male (21/31; 68%), and the majority received a reduced intensity conditioning (RIC) regimen (27/31; 87%). Prophylaxis included CyA + Methotrexate (MTX) *n* = 26 (84%), CyA + Mycophenolate Mofetil (MMF) *n* = 2 (6%), and Tacrolimus +/– MTX *n* = 3 (10%). The majority of patients received a matched unrelated donor (MUD) transplant (23/31; 74%), 13/31 (42%) were cytomegalovirus (CMV) positive and 16/31 (52%) patients were alive at the time of last follow up (Table 1). There was no significant difference in age, gender, diagnosis, conditioining, prophylaxis, relation or CMV status between patients who developed aGvHD and those that remained disease-free (Table 1). A higher proportion of patients with aGvHD were deceased at the time of last follow up (*p* = 0.01; Table 1).

**Figure 1:**
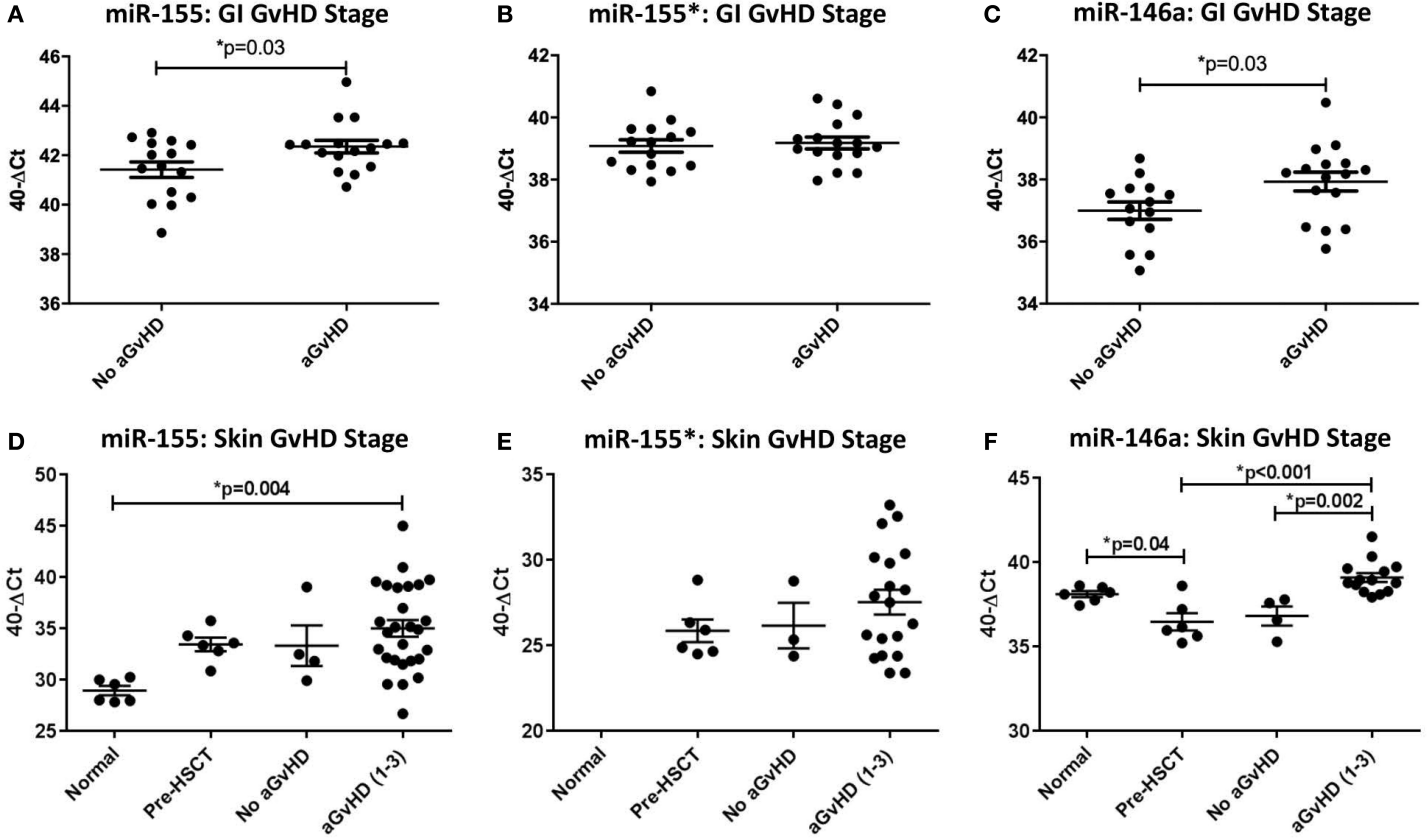
MiR-155, miR-155*, and miR-146a expression in gastrointestinal and skin tissue. MiR-155, miR-155*, and miR-146a expression was assessed in gastrointestinal (GI) and skin biopsies by TaqMan qRT-PCR. **(A–C)** miR-155, miR-155*, and miR-146a expression, respectively, in GI tissue according to GI histopathology aGvHD stage (aGvHD stage 1–4 *n* = 16, no aGvHD stage 0 *n* = 15). **(D–F)** miR-155, miR-155*, and miR-146a expression, respectively, in skin tissue according to skin histopathology aGvHD stage (aGvHD stage 1–3 *n* = 27, no aGvHD stage 0 *n* = 4), normal controls (*n* = 6), and pre-HSCT biopsies (*n* = 6). Significance between groups was calculated using the independent *t*-test or one-way ANOVA with Tukey *post-hoc* multiple comparisons adjustment. Error bars shown mean with SEM. * = Significant *p*<0.05.

When assessed according to Lerner grading system, miR-155 was expressed at a significantly higher level in GI aGvHD samples (stage 1–4, *n* = 16) at the time of onset compared to no GvHD (stage 0, *n* = 15) (*p* = 0.03; Figure 1A). There was no significant difference in miR-155^*^ expression when assessed according to GI aGvHD incidence (Figure 1B). Expression of miR-146a was also higher in patients with GI aGvHD compared to patients who did not develop GvHD (*p* = 0.03; Figure 1C). In relation to clinical outcome, expression of neither miR-155 nor miR-146a was significantly associated with OS following HSCT (*p* > 0.05).

### MiR-155, miR-155^*^, and miR-146a Expression in Skin Tissue

Skin biopsies were taken from *n* = 31 HSCT patients at the time of clinical aGvHD onset [aGvHD *n* = 27 (87%, mean aGvHD onset 41 days post-HSCT): stage 1 = 19 (61%), 2 = 6 (19%), and 3 = 2 (7%); no aGvHD *n* = 4 (13%)] (Table 1), pre-HSCT day 0 (*n* = 6) or from normal healthy volunteers (*n* = 6). Patients were of mixed underlying diagnosis, the majority were male (24/31; 77%), conditioning comprised of predominantly RIC (25/31; 81%) and all patients received CyA + MTX prophylaxis. The majority of patients received MUD transplants and 12/31 (39%) were CMV positive, while 18/31 (58%) were alive at the time of last follow up (Table 1). There was no significant difference in age, gender, diagnosis, conditioining, prophylaxis or relation between patients who developed aGvHD and those that remained disease-free (Table 1), however, a higher proportion of patients with aGvHD were CMV positive (*p* = 0.02).

MiR-155, miR-155^*^, and miR-146a expression was examined in normal control skin, pre-transplant skin and skin samples taken at aGvHD onset (*n* =43). In relation to aGvHD incidence, based on cutaneous histopathology grading, expression of miR-155 was significantly up-regulated in aGvHD (stage 1–3) compared to normal skin (*p* = 0.004; Figure 1D). Overall, miR-155 expression was not associated with aGVHD severity or post-HSCT OS. MiR-155^*^ was not expressed in normal skin (*n* = 6). However, expression was detected in skin samples taken at aGvHD onset in 22/31 (71%) of the patients. MiR-155^*^ was not associated with aGvHD severity or OS (Figure 1E). Expression of miR-146a was significantly higher in patients with skin aGvHD (stage 1–3) compared to no skin aGvHD (*p* = 0.002; Figure 1F). Skin samples taken at aGvHD onset (stage 1–3) also had higher expression of miR-146a compared to pre-HSCT samples (*p* < 0.001). Expression of miR-146a was significantly higher in normal controls compared to pre-HSCT (*p* = 0.04; Figure 1F). MiR-146a expression was not associated with OS.

### MiR-155, miR-155^*^, and miR-146a Expression in Serum

Expression of miR-155, miR-155^*^, and miR-146a was assessed in an exploratory cohort of sequential serum samples taken from patients pre-HSCT, at the time of HSCT (D0) and D7, D14 and D28 post-HSCT (*n* = 34) to investigate expression patterns during early transplant (Table 2). Of the 34 patients, *n* = 20 (59%) developed clinical overall aGvHD [mean aGvHD onset 44 days post-HSCT, grades I = 6 (18%), II = 8 (23%) and III = 6 (18%)], while *n* = 14 (41%) remained disease-free (Table 2). Patients were of mixed underlying diagnosis, the majoriy were male (25/34; 74%), received a RIC regimen (28/34; 82%), and a MUD transplant (27/34; 79%). A total of 15/34 (44%) patients were CMV positive and 18/34 (53%) were alive at the time of last follow up (Table 2). Prophylactic therapy comprised of Cyclosporine A for all patients. There was no signficiant difference in age, gender, conditioning, relation, or survival between patients who developed aGvHD and those that remained disease-free, however, there was a slightly higher proportion of CMV positive patients in the GvHD-free group (*p* = 0.04; Table 2).

**Figure 2:**
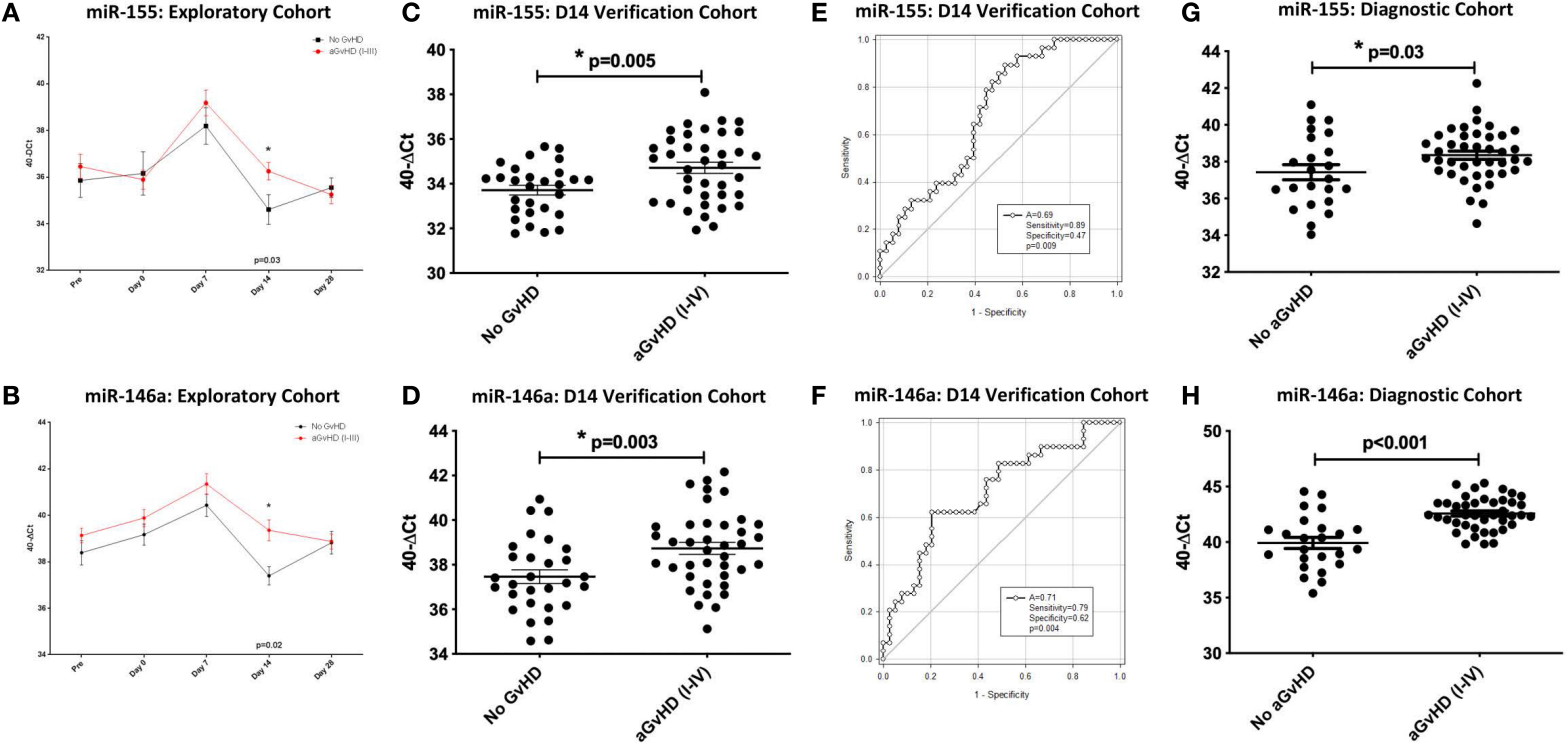
MiR-155 and miR-146a expression in serum. MiR-155 and miR-146a expression was assessed in serum cohorts by TaqMan qRT-PCR. **(A,B)** Differential miR-155 and miR-146a expression, respectively, in serum samples of the sequential exploratory cohort (*n* = 34) time points, segregated based on the incidence of overall aGvHD. Error bars represent mean with SEM and p-values were calculated using the independent *t*-test. **(C,D)** MiR-155 and miR-146a expression, respectively, at D14 in the verification cohort (*n* = 81) according to aGvHD incidence. Box plot whiskers represent minimum to maximum expression and p-values were calculated using the independent *t*-test. **(E,F)** Receiver operator analysis of miR-155 and miR-146a expression, respectively, in relation to incidence of aGvHD in the extended D14 serum verification cohort (*n* = 81). **(G,H)** MiR-155 and miR-146a expression, respectively, at aGvHD onset in the diagnostic cohort (*n* = 65) according to overall aGvHD incidence. Box plot whiskers represent minimum to maximum expression and *p*-values were calculated using the independent *t*-test. * = Significant *p*<0.05.

In relation to clinical overall aGvHD incidence, there was significantly higher expression of miR-155 in patients who subsequently developed aGvHD (grade I–IV) compared to those who remained disease-free (grade 0) at D14 (*p* = 0.03) in the exploratory cohort (Figure 2A). In addition, miR-155 expression in aGvHD patients was elevated at D7 compared to at the time of transplant (D0) or at D14 post-HSCT time points (D7 vs. D0 aGvHD *p* < 0.01; D7 vs. D14 aGvHD *p* < 0.01; Supplementary Figure 1). In contrast, miR-155^*^ expression was not detected in the sera (data not shown). Expression of miR-146a was significantly higher in patients who subsequently developed clinical overall aGvHD at all time points analyzed, and this difference was significant at D14 (*p* = 0.02; Figure 2B). The level of miR-146a expression in aGvHD patients was highest at D7 in comparison to D0, and levels had significantly decreased by D14 (D7 vs. D0 aGvHD *p* = 0.03; D7 vs. D14 noaGvHD *p* = 0.04; Supplementary Figure 1).

In order to verify miR-155 and miR-146a data, expression was investigated in an extended verification cohort of sera taken at D14 post-HSCT (*n* = 81), prior to the onset of symptomatic aGvHD (Table 2). The cohort comprised of *n* = 44 (54%) patients who developed clinical overall aGvHD [mean onset 42 days post-HSCT: grades I = 5 (6%), II = 31 (38%), and III = 8 (10%)] and *n* = 37 (46%) patients who remained aGvHD free (Table 2). Patients were of mixed underlying diagnosis, a slight majority were male (48/81; 59%) and predominantly received a RIC transplant (61/81; 75%) from a MUD donor (47/81; 58%). Data on CMV status was available for 76/81 (94%) patients, of which 37/76 (49%) were CMV positive (Table 2). Prophylactic therapy comprised of Cyclosporine A for all patients. At the time of follow up, *n* =16 patients were relapsed or deceased, *n* = 29 had non-relapse mortality (NRM) and *n* =35 were in remission. There was no significant difference in age, gender, conditioning, relation or survival in patients who developed aGvHD compared to those that remained disease-free (Table 2). A higher proportion of patients that developed aGvHD were CMV-negative (*p* = 0.01; Table 2). Expression of both miR-155 (*p* = 0.005) and miR-146a (*p* = 0.003) was higher in patients who subsequently developed overall aGvHD compared to those that remained GvHD-free (Figures 2C,D). Both microRNAs were also significantly associated with developing aGvHD by ROC analysis, whereby the optimal cutoff value to dichotomise microRNA expression was computed from sensitivity and specificity (28) (miR-155 AUC = 0.69, sensitivity = 0.89, specificity = 0.47, *p* = 0.009; miR-146a AUC = 0.71, sensitivity = 0.79, specificity = 0.62, *p* = 0.009; Figures 2E,F). When patients were grouped according to no/mild clinical GvHD (grade 0–I vs. II–IV), expression of miR-146a was higher in patients with severe GvHD (grade II–IV) compared to patients with no/mild aGvHD (grade 0–I) (*p* = 0.003), while expression of miR-155 showed a trend to higher expression in patients with severe aGvHD (stage II–IV) (*p* = 0.07). Neither miR-146a nor miR-155 were significantly associated with OS by ROC analysis or by Kaplan-Meier and Log Rank test, when microRNA values were assessed for their ability to discriminate between those that were dead and those that were alive (*p* > 0.05). The expression of miR-155 and miR-146a in the verification cohort was significantly correlated (*p* < 0.001, *R*^2^ = 0.21).

To further explore miR-155 and miR-146a, expression was assessed in an independent diagnostic cohort (*n* = 65) of samples from a separate Institution (Vienna) taken at onset of symptoms and diagnosis of overall aGvHD, or at corresponding time points in patients who did not develop aGvHD (Table 2). The cohort comprised *n* = 65 HSCT patients [aGvHD *n* = 41 (63%) (mean onset 34 days post-HSCT: grades I = 22 (34%), II = 4 (6%) and III = 15 (23%)), no aGvHD *n* = 24 (37%)] (Table 2). Patients were of mixed underlying diagnosis, a slight majority were male and received a RIC transplant (32/57; 56%) from a MUD donor (43/57; 75%) (Table 2). CMV status as available for 44/65 (68%) patients, of which 27/44 (61%) were CMV positive. At the time of last followup, 8/65 (12%) patients were deceased (Table 2). Prophylactic therapy information was available for 53/65 (82%) patients (Cyclosporine A (CyA) + Mycophenolate Mofetil (MMF) *n* = 24 (45%), CyA + Methotrexate (MTX) *n* =19 (36%), MTX *n* = 7 (13%), MMF *n* = 2 (4%), CyA *n* = 1 (2%). There was no significant difference in age, gender, conditioning, relation, CMV status, or survival between patients who developed aGvHD compared to those that remained disease-free (Table 2). Expression of miR-155 (*p* = 0.03) and miR-146a (*p* < 0.001) was significantly higher in patients with overall clinical aGvHD at the onset of symptoms, compared to patients with no aGvHD (Figures 2G,H). Expression was not associated with OS by ROC analysis or by Log Rank test (*p* > 0.05). When assessed according to severity, there was no association between miR-146a or miR-155 and patients with severe (grade II–IV) disease compared to no/mild aGvHD (grade 0–I) (*p* > 0.05).

### MiR-155, miR-155^*^, and miR-146a Expression in Serum Extracellular Vesicles

**Figure 3:**
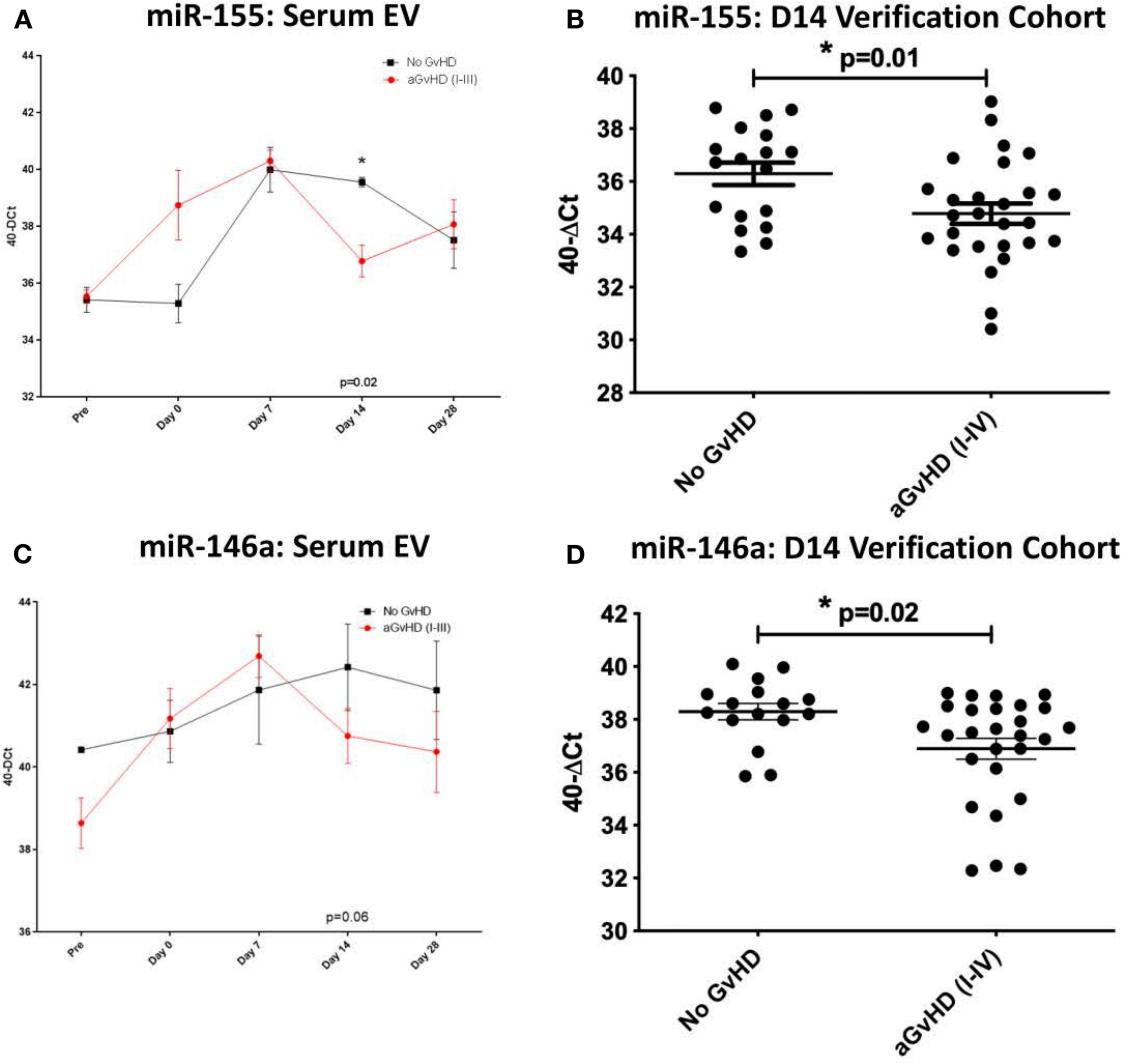
MiR-155 and miR-146a expression in serum extracellular vesicles. MiR-155 and miR-146a expression was assessed in the serum extracellular vesicle fraction by TaqMan qRT-PCR. **(A,B)** MiR-155 and miR-146a expression, respectively, in the EV fraction of serum samples of the exploratory cohort, taken at sequential time points pre- and post-HSCT (*n* = 15) analyzed according to the incidence of overall aGvHD. Differential serum EV microRNA expression between patients who developed overall aGvHD vs. those who remained disease-free was assessed and significant or borderline differences are shown. Error bars represent mean with SEM and p-values were calculated using the independent *t*-test. **(C,D)** MiR-155 and miR-146a expression, respectively, in the EV fraction of the D14 verification cohort (*n* = 47) according to aGvHD incidence. Box plot whiskers represent minimum to maximum expression and *p*-values were calculated using the independent *t*-test. * = Significant *p*<0.05.

MiR-155, miR-155^*^, and miR-146a expression was further explored within the EV fraction of serum, from a subset of patients (*n* = 15) of the sequential time point exploratory cohort previously described (Table 2). At D14 post-HSCT, prior to the onset of aGvHD, miR-155 was significantly down-regulated in serum EV of patients who subsequently developed aGvHD compared to those that remained disease-free (*p* = 0.02; Figure 3A). MiR-155^*^ expression was not detected in serum EVs in the sequential exploratory cohort and thus, was excluded from further analysis. Similar to miR-155, miR-146a expression in serum EVs showed a trend toward lower expression at D14 in patients who subsequently developed aGvHD compared to those who did not (*p* = 0.06; Figure 3B). Downregulated miR-155 (*p* = 0.01) and miR-146a (*p* = 0.02) expression in serum EVs at D14 was further confirmed using *n* = 47 samples of the independent verification cohort (*n* = 65) (Table 2) of serum EV samples taken at D14 post-HSCT (Figures 3C,D).

### miR-155, miR-155^*^, and miR-146a Expression in Urine

MiR-155, miR-155^*^ and miR-146a expression was assessed in an exploratory cohort of urine samples by qRT-PCR at sequential time points from pre-HSCT to D14 post-HSCT (Table 3). The cohort comprised *n* = 30 HSCT patients [aGvHD *n* = 21 (70%) mean onset 56 days post-HSCT: grades I = 9 (30%), II = 11 (37%), and III = 1 (3%), no aGvHD *n* = 9 (30%)] (Table 3). Patients were of mixed underlying diagnosis, the majority were male (19/30; 63%) and received a myeloablative transplant from a MUD donor (20/30; 67%) (Table 3). A total of 17/30 (57%) patients were CMV positive and 19/30 (63%) were alive at the time of last follow up (Table 3). There was no significant difference in age, gender, conditioning, relation, CMV status or survival between patients who developed aGvHD compared to those that remained disease-free (Table 3).

**Figure 4:**
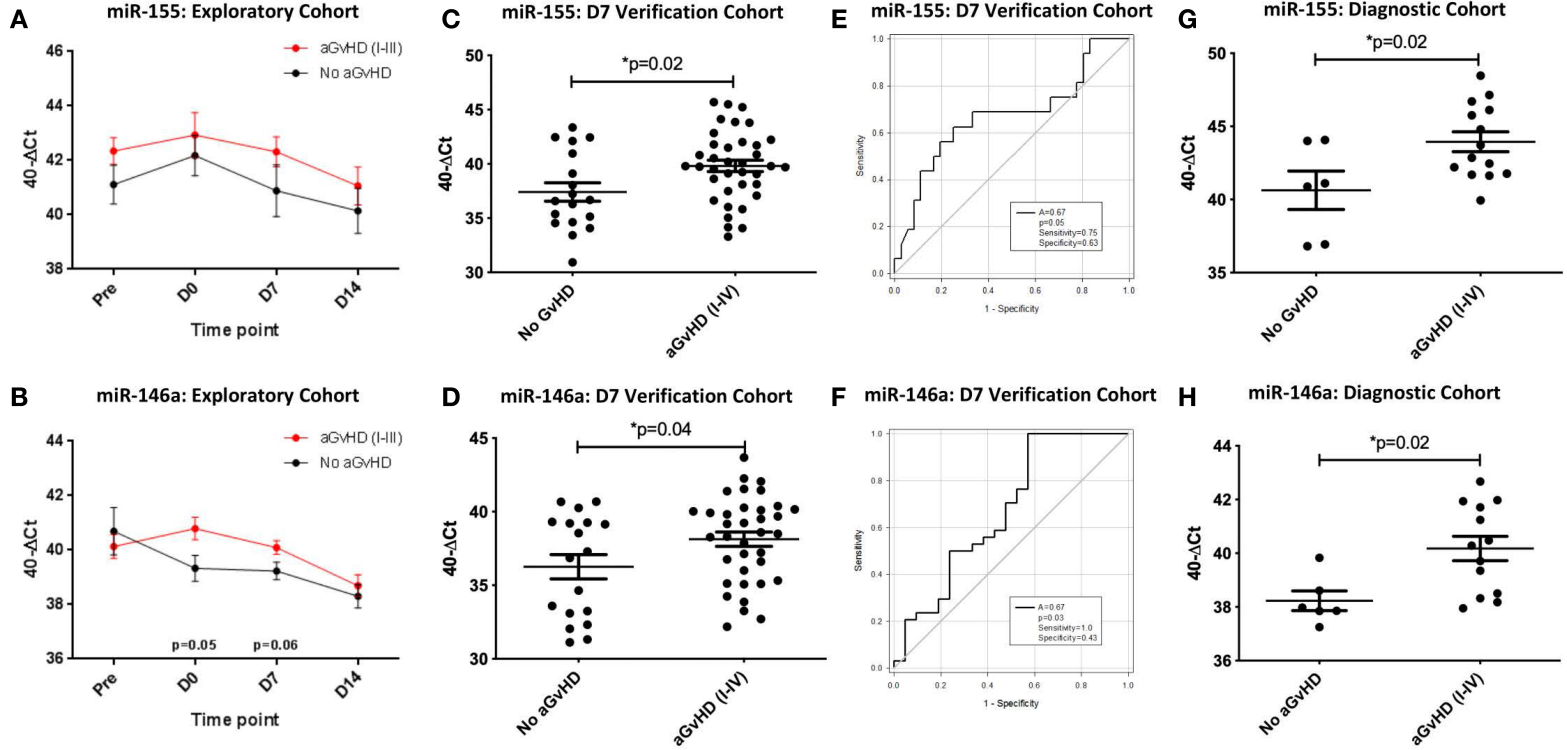
miR-155 and miR-146a expression in urine. MiR-155 and miR-146a expression was assessed in urine by TaqMan qRT-PCR. **(A,B)** MiR-155 and miR-146a expression, respectively. Patients of the exploratory cohort (*n* = 30) were segregated based on the incidence of aGvHD and microRNA expression plotted at each sequential time point. Differential microRNA expression between patients who developed overall aGvHD vs. those who remained disease-free was assessed and significant or borderline differences are shown. Error bars represent mean with SEM and *p*-values were calculated using the independent *t*-test. **(C,D)** MiR-155 and miR-146a expression, respectively, in the extended D7 urine sample verification cohort (*n* = 56) according to aGvHD incidence. Box plot whiskers represent minimum to maximum expression and p-values were calculated using the independent *t*-test. **(E,F)** Receiver operator analysis of miR-155 and miR-146a expression, respectively, in relation to incidence of aGvHD in the extended D7 urine sample verification cohort (*n* = 56). **(G,H)** MiR-155 and miR-146a expression, respectively, at aGvHD onset in the urine diagnostic cohort (*n* = 20) according to aGvHD incidence. Box plot whiskers represent minimum to maximum expression and *p*-values were calculated using the independent *t*-test. * = Significant *p*<0.05.

Expression of miR-155 showed a trend toward higher expression in patients who subsequently developed clinical overall aGvHD (grade I–IV), though this did not reach significance (Figure 4A). In contrast, expression of miR-155^*^ was not detected in urine samples (data not shown). Expression of miR-146a also showed a trend for higher expression in patients who subsequently developed aGvHD at D0 (*p* = 0.05) and D7 (*p* = 0.06; Figure 4B).

An extended cohort of urine samples was further assessed for miR-155 and miR-146a expression prior to aGvHD onset, comprising of urine samples taken at D7 post-HSCT, prior to the onset of symptomatic disease, from *n* = 56 patients who later developed aGvHD (aGvHD *n* = 38 (68%) [mean aGvHD onset 44 days post-HSCT: grades I = 18 (32%), II = 17 (31%), and III = 3 (5%); no aGvHD *n* = 18 (32%)] (Table 3). Patients were of mixed underlying diagnosis, the majority were male (39/56; 70%), received a RIC transplant (42/56; 75%) from a MUD donor (37/56; 66%) and were CMV negative (30/56; 54%) (Table 3). At the time of follow up, *n* = 19 patients were relapsed or deceased, *n* = 10 had NRM and *n* = 27 were in remission. There was no significant difference between patient age, gender, conditioning, relation, CMV status, or survival between patients who developed aGvHD compared to those that remained disease-free (Table 3). Expression of both miR-155 (*p* = 0.02) and miR-146a (*p* = 0.04) was significantly higher in patients who subsequently developed overall aGvHD (grade I–IV) compared to those that remained disease free (Figures 4C,D). Both microRNAs showed prognostic potential for aGvHD by ROC analysis (miR-155 AUC = 0.67, sensitivity = 0.75, specificity = 0.63, *p* = 0.05; miR-146a AUC = 0.67, sensitivity = 1.00, specificity = 0.43, *p* = 0.03; Figures 4E,F). Neither miR-155 nor miR-146a were significantly associated with OS by ROC or Log Rank analysis (*p* > 0.05). When microRNA expression was analyzed in relation to aGvHD severity, there was no significant difference in miR-146a or miR-155 expression in patients who developed severe aGvHD (II–IV) compared to those that had mild or no aGvHD (0–I) (*p* > 0.05). The expression of miR-155 and miR-146a at D7 was significantly positively correlated (*p* < 0.001, *R*^2^ = 0.62).

To further explore the biomarker potential of miR-155 and miR-146a, expression was assessed in a diagnostic cohort (*n* = 20) of urine samples taken at the onset of aGvHD symptoms, from an independent cohort of patients transplanted in a separate transplant center (Regensburg) (diagnostic cohort, *n* = 20) (Table 3). Patients were of mixed underlying diagnosis, approximately half of patients were male (11/20), the majority received a RIC transplant (14/20; 70%) from a MUD donor (14/20; 70%) and were CMV negative (13/20; 65%) (Table 3). There was no significant difference between patient age, gender, conditioning, relation, or CMV status beteen patients who developed aGvHD compared to those that remained disease-free (Table 3). Expression of miR-155 (*p* = 0.02) and miR-146a (*p* = 0.02) was higher in patients with aGvHD (stage I–IV) compared to those who did not have the disease (Figures 4G,H), indicating these microRNAs to demonstrate altered expression at aGvHD onset. When analyzed according to aGvHD severity, expression of miR-155 (*p* = 0.006) and miR-146a (*p* = 0.008) was significantly higher in patients with severe aGvHD (stage II–IV) compared to no/mild aGvHD (stage 0–I).

### miR-155, miR-155^*^, and miR-146a Expression in Urine Extracellular Vesicles

Expression of miR-155, miR-155^*^, and miR-146a was also assessed in the EV fraction of urine in an exploratory cohort of sequential samples taken from pre-HSCT to D14 post-HSCT (Table 3).

**Figure 5:**
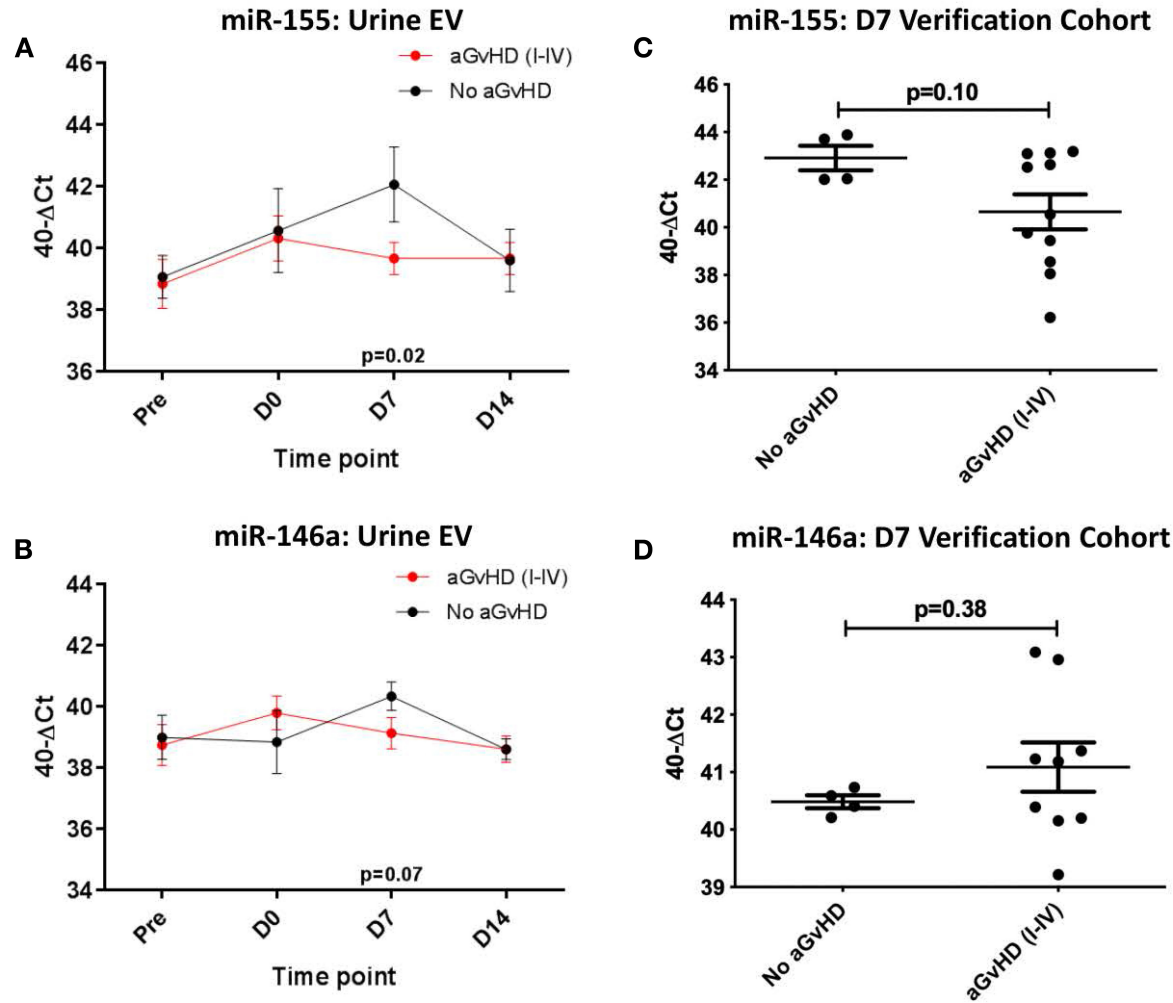
miR-155 and mIR-146a expression in urine extracellular vesicles. MiR-155 and miR-146a expression was assessed in urine extracellular vesicles by TaqMan qRT-PCR. **(A,C)** MiR-155 and miR-146a expression, respectively. Patients of the sequential cohort (*n* = 26) were segregated based on the incidence of aGvHD and microRNA expression in EVs was plotted at each time point. Differential microRNA expression between patients who developed aGvHD vs. those who remained disease-free was assessed and significant or borderline differences are shown. Error bars represent mean with SEM and *p*-values were calculated using the students *t*-test. **(B,D)** MiR-155 and miR-146a expression, respectively. Expression at D7 in EVs in the verification cohort (*n* = 15) according to aGvHD incidence. Box plot whiskers represent minimum to maximum expression and *p*-values were calculated using the independent *t*-test.

In the sequential exploratory cohort (*n* = 30) (Table 3), miR-155 expression was significantly lower at D7 in patients who subsequently developed aGvHD compared to patients that did not (*p* = 0.02; Figure 5A). Expression of miR-155^*^ was not detected in the urine EV samples, regardless of time point or aGvHD grade (data not shown). Expression of miR-146a in the sequential exploratory cohort was similar in patients who developed aGvHD compared to those who did not, with the exception of the D7 time point where miR-146a showed a trend to lower expression in patients who remained aGvHD-free, although not to significance (*p* = 0.07; Figure 5B).

Expression of miR-155 and miR-146a was further assessed at D7 in urine EVs of a small independent cohort of samples (*n* = 15). Expression of miR-155 (*p* = 0.10) showed a trend toward lower expression in patients whoe developed aGvHD, while there was no significant difference in expression of miR-146a (Figures 5C,D).

## Discussion

This study analyzed the expression of miR-155, miR-155^*^, and miR-146a in target tissues (normal skin, aGvHD skin and aGvHD gastrointestinal tissue) and body fluids (serum, urine and their EV components) affected by aGvHD from patients undergoing HSCT. MicroRNAs have been shown to be important in the control of immunity, and miR-155 and miR-146a function in immunoregulation by modulating both the adaptive and innate immune response (11, 17). Although both miR-155 and miR-146a have previously been reported to play a role in aGvHD (11, 17, 18), to date their expression has not been comprehensively profiled in aGvHD target organs, or in biofluids and EVs from post-HSCT patients.

Ranganathan et al. were the first to report an association between miR-155 and aGvHD, including strong up-regulation of miR-155 expression in the inflammatory cells of all patients with small- and large-bowel aGvHD, whereas miR-155 expression was absent in normal bowel (11). In the present study, we assessed miR-155 expression in *n* = 31 GI biopsies taken from patients with or without GI GvHD and observed significantly elevated levels of miR-155 in patients who were diagnosed with GI aGvHD (stage 1–4), in agreement with Ranganathan et al. (11). In GI tissue, miR-155 has previously been reported to play an important role in intestinal mucosal barrier (IMB) function, where it regulates ras homolog family member A (RhoA), resulting in downregulation of major protein components of the apical junction complex of the IMB (29). IMB function is closely associated with intestinal disease, such as irritable bowel disease (IBD), and miR-155 expression has also shown to be directly elevated in IBD such as ulcerative colitis and Chrohn's disease (30, 31). In GvHD, factors such as LPS and other bacterial products that are important in GvHD pathogenesis may undergo increased influx in the presence of increased intestinal permeability, partly caused by miR-155. Although the precise molecular mechanisms behind miR-155 function in intestinal disease and inflammation are not fully understood, the most documented pathway is via control of cytokine release (32). With regard to miR-146a, expression was also elevated in the GI tissue of patients with aGvHD. This is discordant with a recent study by Gartner et al. who showed an increase in intestinal permeability on day 15 in a mouse model of GvHD, which corresponded to reduced miR-146a expression (18). This is an interesting observation, as LPS has been shown to induce miR-146a expression in human monocytic leukemia cells (16), via binding to TLR4, triggering an intracellular signaling pathway that results in activation of NF-κB and subsequent miR-146a upregulation (33, 34). While the studies of Gartner et al. focussed on day 15, the present study patient cohort was comprised of GI tissue samples taken at the onset of clinical aGvHD symptoms. The balance between LPS influx, tissue permeability and miR-146a expression in GvHD target GI tissue warrants further investigation to fully understand the mechanistic pathways at a molecular level during the development of GvHD and timing of symptomatic disease. Although miR-155^*^ was expressed in GI biopsies in the present study, it was not associated with occurance of GI GvHD.

As skin may be considered one of the primary target organs of aGvHD, the role of aGvHD associated microRNAs in a skin-specific environment may further our understanding of the molecular pathology of the disease. In this regard, miR-155 expression was assessed in skin biopsies taken at the onset of aGvHD and we found significant up regulation of miR-155 in aGvHD skin with a high histology aGvHD stage (stage 1–3), in comparison to normal controls. This is in accordance with our previous microRNA profiling studies where we found elevated miR-155 expression in the skin of a rat GvHD model (35), as well as in cutaneous GvHD, whereby miR-155 expression was elevlated at the time of GvHD onset compared to healthy control skin (36). However, in the present study although expression was higher in a proportion of patients with aGvHD compared to those with no skin aGvHD, or pre-HSCT biopsies, this did not reach significance. This suggests that in the skin, miR-155 expression may be affected by the HSCT conditioning regimen triggering inflammatory skin damage, that is not specifically associated to aGvHD. Indeed, miR-155 has been found to be highly up-regulated by infiltrating immune cells in the skin of patients with atopic dermatitis (37). In this context, miR-155 is upregulated during T-cell differentiation and activation, as well as by allergens. Down regulation of cytotoxic T-lymphocyte associated protein 4 (CTLA-4), a negative regulator of T-cell activation and a target of miR-155 could contribute to the skin inflammation observed (37). Although the present study focused on miR-155 expression, we did not assess expression of its potential targets in the skin, which would be informative to evaluate in future studies. MiR-155^*^ was not expressed in normal control skin samples, but was expressed in skin samples taken at suspected aGvHD onset in 71% of the patients. However, this expression did not correlate with aGvHD incidence or severity according to either clinical or histology stage. Overall, this suggests that miR-155^*^ expression is triggered by the HSCT conditioning or procedure, but is not associated with skin aGvHD. Deep sequencing of murine microRNAs has shown that tissue specific expression differences exist between miR-5p or miR-3p strands (38). It is entirely possible therefore that expression of miR-155^*^ is tissue and inflammation specific, as we did find low levels of miR-155^*^ expression in some of the skin biopsies taken at onset of GvHD, but there was no expression in normal or pre-transplant skin samples. MiR-146a expression was significantly higher in samples with a high skin histology aGvHD stage (stage 1–3) compared to patients with no skin aGvHD, and compared to the pre-HSCT group. We have previously associated expression of miR-146a with development of GvHD in a rat model (35), and miR-146a has known roles in skin inflammation and inflammatory conditions such as psoriasis and atopic dermatitis (39–41). In psoriasis, miR-146a is significantly over-expressed in psoriatic lesions compared with healthy control skin. Expression levels were highest in organs containing leukocytes and showed low expression in healthy skin, suggesting the high levels observed in psoriasis were due to infiltrating cells (40). Expression of miR-146a is regulated by the transcription factor nuclear factor-Kβ (NF- Kβ) and interestingly, two of its validated targets TRAF6 and IRAK1 (16) are TNF-α regulators, suggesting that miR-146a may control TNF-α signaling in the skin (42). The elevated miR-146a levels observed in the skin of aGvHD patients in the present study support further investigation of the role of miR-146a targets, specific to skin tissue. The hypothesis that elevated expression observed in clinical aGvHD skin biopsies may be in part due to infiltrating cells that highly express miR-146a also warrants consideration.

Focusing on bodily fluids, we found significantly higher expression of serum miR-155 and miR-146a in patients who subsequently developed overall clinical aGvHD (grade I–IV) compared to those who remained aGvHD-free (grade 0) in samples taken at D14 post-HSCT. This result was verified in an independent cohort of transplant samples taken at the onset of symptoms, and both microRNAs were found to be significantly up upregulated in patients with overall aGvHD (grade I–IV) compared to patients with no aGvHD. Conversely, in serum EV samples we found significant downregulation of miR-155 and miR-146a expression at D14. In urine samples taken on D7 post-HSCT, the expression of both miR-155 and miR-146a was significantly higher in patients who subsequently developed clinical aGvHD (grade I–IV) compared to patients who remained aGVHD-free. Similarly, the expression of both miR-155 and miR-146a in D7 post-HSCT urine EV samples was higher in patients who did not develop aGvHD, although not to significance. We found no evidence of miR-155^*^ expression in serum or urine (including their EV compartments).

Although expression of miR-155 and miR-146a in body fluids may be informative from an aGvHD biomarker perspective, it is challenging to elucidate the source of expression of these microRNAs. Further studies are required in order to understand their cell of origin as well as functionality in aGvHD pathobiology at a systemic level, given their complex roles in immunity and inflammation. Indeed, elevated expression of miR-155 in the serum of aGvHD patients may be directly related to its central role of miR-155 in inflammation, where it has been identified as a component of the primary macrophage response to inflammatory mediators such as LPS, IFN-β, polyriboinosinic-polyribocytidylic acid (poly IC), and TNF-α (43). Furthermore, up-regulated miR-155 expression is associated with increased cytokine release during the inflammatory response (44). However, miR-155 has also been found to control the intensity of the inflammatory response by targeting the Toll-like receptor/interleukin-1 (TLR/IL-1) inflammatory pathway in human dendritic cells (DCs) (45). Indeed, miR-155 is important in the regulation of myeloid cells, where it is required for optimum DC production of cytokines (45). Induction of miR-155 expression in DC- exposed to tol-like receptor 4 (TLR4) ligand and LPS leads to modulation of the IL-1 signaling pathway (45). Ceppi et al. therefore proposed that miR-155 functions as part of the negative feedback loop controlling the secretion of inflammatory cytokines by LPS-induced DC activation and thus, is pivotal in the fine tuning of the immune response (45). MiR-155 is also up regulated during T-cell differentiation and promotes the development of T-cells, including Th17 and regulatory T-cell (Treg) subsets (46). MiR-155 has been previously implicated in the pathogenesis of autoimmune diseases, including rheumatoid arthritis (RA) (47) and systemic lupus erythematosus (SLE) (48) as well as in aGvHD, where in a murine model miR-155 is upregulated in T-cells and regulates the severity of aGvHD, indicating a pro-inflammatory role of miR-155 (11). Subsequent studies assessing miR-155 expression as a biomarker for aGvHD in plasma and serum samples have shown promise (12, 13), and biofluids as sources of biomarkers in general are advantageous, as sample collection in non-invasive.

MicroRNA-146 also plays a key role in the regulation of innate as well as adaptive immunity and we have previously associated high serum expression post-HSCT with patients who subsequently develop aGvHD (19). MiR-146a has been shown to target IRAK1 and TRAF6, and functions as a negative regulator in Toll-like receptor (TLR) and pro-inflammatory cytokine (IL-1) signaling pathways (16, 17). IRAK1 codes for a key intracellular signaling protein that is activated by ligands of TLRs. IRAK1 links TLR with the TRAF6 intracytoplasmic activator of transcription factor NF-κB, which subsequently increases the expression of a number of genes related to immunological response such as TNF-α and IL-8 (49, 50). Subsequently, IRAK1 is subjected to negative feedback by miR-146a, the expression of which is also NF-κB dependent, leading to a concerted immunological response. MiR-146a is highly expressed in Treg cells and is induced upon activation of effector T-cells and myeloid cells. In the latter, miR-146a acts as a negative feedback regulator to limit TRAF6 and IRAK1-mediated signaling in an inflammatory settings (16, 51), whereas in activated human T cells, miR-146a has been suggested to oppose apoptosis and IL-2 production (52). With regard to GvHD, LPS can trigger miR-146a expression (16, 17). Upon stimulation with LPS or monocyte activation via cell surface receptors such as TLR4, miR-146a will target *IRAK1* and *TRAF6* both *in vivo* and *in vitro* and is partially responsible for IL-1-induced upregulation of NF-kB (53). Stickel et al. showed that transfer of miR-146a-deficient T-cells caused increased GVHD severity, elevated TNF-α serum levels, and reduced survival (17). In addition, TRAF6 was increased in miR-146a-deficient T-cells upon contact with alloantigen, which translated into increased nuclear factor-κB activity and TNF-α production in miR-146a-deficient T-cells. In contrast, the use of a miR-146a mimic reduced aGvHD severity (17).

It was interesting that the EV compartment of serum demonstrated lower expression of miR-155 and miR-146a expression at D14 post-HSCT in patients who developed aGvHD, and suggests specific packaging of these microRNAs in response to post-HSCT processes. Interestingly, both microRNAs demonstrated increased expression in patients who later developed aGvHD on D0 and D7, which then decreased dramatically by D14 compared to patients who remained aGvHD-free. In a mouse model system, Alexander et al. have shown that the EVs of bone marrow derived DCs contain miR-155 that can reprogramme recipient DCs in a manner that enhances their response to LPS, while miR-146a dampens the pro-inflammatory response by DCs following LPS treatment (54). These results are consistent with previous observations that miR-155 and miR-146a play opposing roles during inflammation. In a transplant setting, as previously described, both miR-155 and miR-146a can be induced by LPS, which is a central component in triggering aGvHD pathology. A recent study by Schulte et al. (55) proposes that in macrophages miR-146a and miR-155 are responsive to different levels of LPS and control different aspects of the TLR4 response. They demonstrated a “checkpoint” effect, where, in an inflammatory response to LPS miR-146a is initially up-regulated. However, it becomes saturated with increasing levels of LPS and as a result, miR-155 is upregulated. Our data suggests a further role for miR-146a and miR-155 in the EVs of post-HSCT patients, however, it is unclear why expression of both microRNAs is downregulated in the EVs of patients who develop aGvHD, particularly in relation to their opposing roles in inflammation. Alexander et al. proposed several possible explanations as to why EVs may contain both of these functionally distinct miRNAs. First, EVs may transfer both pro- and anti-inflammatory miRNAs together in order to counteract inflammatory responses by recipient cells. Second, it is possible that miR-155 and miR-146a are encapsulated into separate EVs that have been assessed collectively as one population, and that individual EVs are delivered to different target cells. Thirdly, release of either microRNA to target cells may be finely tuned in a dynamic manner, whereby the ratio of each microRNA release can change over time (54). Overall, our data further suggest a role for EV miR-155 and miR-146a in aGvHD pathology that requires further investigation.

Although we have included verification cohorts to validate the results obtained from body fluid samples, verification GI and skin tissue cohorts are challenging to compile. Despite our results being consistent with previous reports, this highlights the need for collaboration amongst the GvHD research community in order to generate well-characterized, multi-center patient cohorts to allow for more extensive verification of potential biomarkers. This is essential not only for comprehensive validation and translation of results into clinical trials, but also for the purpose of collating detailed clinical information to evaluate the homogeneity of patient cohorts. In the present study, across all cohorts, the majority of patients were male, received RIC conditioning with Cyclosporine A (+MTX/MMF) prophylaxis and a MUD donor. Patient CMV status was varied, whereby some cohorts had a majority of CMV positive patients, while others were mainly CMV negative. Although it has been shown that CMV infection can alter the expression of some cellular microRNA (56), there was no significant difference in miR-146a, miR-155^*^, or miR-155 expression between patients CMV positive compared to CMV negative for any of the cohorts (data not shown). Another limitation of the study is highlighted by the limited specificity of individual microRNAs by ROC analysis. This is an important consideration, as while we are initially reporting that individual microRNAs are raised in specific patient groups compared to others, the results are too preliminary to claim that these microRNAs can individually act as biomarkers for diagnostic or prognostic purposes. Indeed, this raises a common issue for developing biomarkers for this intent; if biomarkers are to be used for diagnostic/prognostic purposes, it is likely that a signature of biomarkers with significant individual differences between groups will give a more accurate discriminatory score, with an improved combined ROC curve that may be incorporated into predictive/prognostic algorithms.

In conclusion, this is the most comprehensive study conducting miR-155 and miR-146a expression profiling in aGvHD to date. We report that expression of miR-155 and miR-146a is elevated in GI aGvHD compared to patients without GI GvHD, and in the skin of patients with cutaneous GvHD compared to normal control skin (miR-155) and compared to patients pre-HSCT and patients with no skin GvHD (miR-146a). Furthermore, expression of both miR-146a and miR-155 was elevated in the serum and urine of patients with overall aGvHD at D14 prior to the onset of symptomatic disease, and also at aGvHD diagnosis. Finally, expression of miR-146a and miR-155 was lower in the serum and urine EVs of patients with overall aGvHD, suggesting specific packaging of these microRNAs into EVs in response to the post-HSCT environment. Overall, our results further support a role for miR-155 and miR-146a in aGvHD, however, the link between their involvement in generalized inflammation and in specific pathophysiology requires further investigation at a systemic level.

## Data Availability Statement

The raw data supporting the conclusions of this article will be made available by the authors, without undue reservation.

## Ethics Statement

The studies involving human participants were reviewed and approved by Newcastle and North Tyneside Research Ethics Committee (REC Red: 14/NE/1136 and 07/H0906/131), University of Regensburg Ethics Commission (approval no 02/220 and 09/059) and Ethics Committee of the Medical University of Vienna, Austria. The patients/participants provided their written informed consent to participate in this study.

## Author Contributions

RC, JN, and AD designed the study and wrote the manuscript. RC and JN performed microRNA expression analysis in skin, serum, urine, and EV samples and analyzed all data. SG performed microRNA expression analysis in GI biopsies and provided GI clinical data. MJ performed microRNA expression analysis in Vienna samples. KP and CL provided Newcaslte clinical data and advised all statistical analysis. MC, EM-W, EH, and HG provided clinical samples and clinical data. All authors approved the manuscript.

## Conflict of Interest

The authors declare that the research was conducted in the absence of any commercial or financial relationships that could be construed as a potential conflict of interest.
