# Supplementary figures and images for: Profiling Tissue and Biofluid miR-155-5p, miR-155*, and miR-146a-5p Expression in Graft vs. Host Disease
Source: Front Immunol. 2021 Mar 15;12:639171. doi: 10.3389/fimmu.2021.639171 (PMC8005601; doi:10.3389/fimmu.2021.639171)

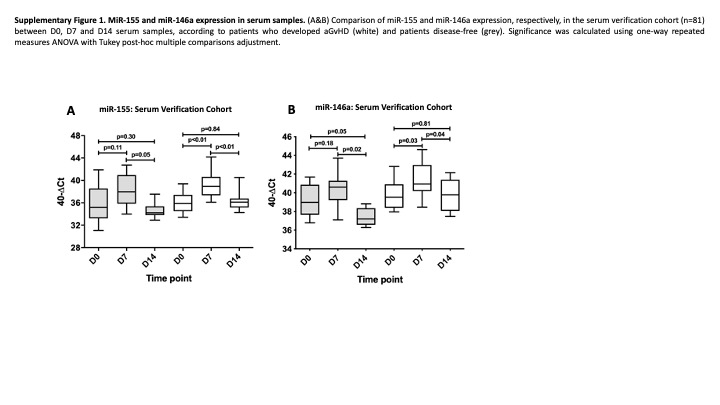

Supplement: Supplementary file 1 [file Image_1.jpeg]
