# Supplementary material for: Profiling Tissue and Biofluid miR-155-5p, miR-155*, and miR-146a-5p Expression in Graft vs. Host Disease
Source: Front Immunol. 2021 Mar 15;12:639171. doi: 10.3389/fimmu.2021.639171 (PMC8005601; doi:10.3389/fimmu.2021.639171)
Supplement: Supplementary file 2 [file Table_1.docx]

| **Sample** | **Reference RNA 1** | **Reference RNA 2** |
| --- | --- | --- |
| Skin | RNU48 | miR-103 |
| Gut | RNU48 | U6 |
| Serum | HY3 | U6 |
| Serum EV | HY3 | U6 |
| Urine | RNU48 | U6 |
| Urine EV | RNU48 | HY3 |

**Supplementary Table 1. Details of endogenous reference RNAs for qRT-PCR experiments.** All endogenous reference controls were identified in previous studies or using NormFinder.
